# Supplementary material for: From sequence to enzyme mechanism using multi-label machine learning
Source: BMC Bioinformatics. 2014 May 19;15:150. doi: 10.1186/1471-2105-15-150 (PMC4229970; doi:10.1186/1471-2105-15-150)
Supplement: Additional file 2 — Java code of ml2db. Additional file ml2db_code.tar.gz contains the Java source code to run the multi-label machine learning experiments and save the results to database. The code’s Javadoc is included. [file 1471-2105-15-150-S2.zip › additional file 2/ml2db/ml2db/doc/mulan/classifier/meta/package-summary.html]

mulan.classifier.meta


JavaScript is disabled on your browser.


- Overview
- Package
- Class
- Use
- Tree
- Deprecated
- Index
- Help

- Prev Package
- Next Package

- Frames
- No Frames

- All Classes

# Package mulan.classifier.meta

- Class Summary

  | Class | Description |
  | --- | --- |
  |  |  |
  | --- | --- |
  | ClusteringBased | Class implementing clustering-based multi-label classification. |
  | ConstrainedKMeans | Cluster data using the constrained k means algorithm |
  | ConstrainedKMeans.bucketInstance | Class for representing an instance inside a bucket |
  | EnsembleOfSubsetLearners | A class for gathering several different SubsetLearners into a composite ensemble model. |
  | HierarchyBuilder | Class that builds a hierarchy on flat lables of given mulltilabel data. |
  | HMC | Class that implements a Hierarchical Multilabel classifier (HMC). |
  | HMCNode | Class that implements a node for the `HMC` |
  | HOMER | Class implementing the Hierarchy Of Multi-labEl leaRners algorithm. |
  | MultiLabelMetaLearner | Base class for multi-label learners, which use other multi-label learners |
  | RAkEL | Class implementing a generalized version of the RAkEL (RAndom k-labELsets) algorithm. |
  | RAkELd | Class implementing a generalized version of the RAkEL-d (RAndom k-labELsets) algorithm with disjoint labelsets. |
  | SubsetLearner | A class for learning a classifier according to disjoint label subsets: a multi-label learner (the Label Powerset by default) is applied to subsets with multiple labels and a single-label learner is applied to single label subsets. |
- Enum Summary

  | Enum | Description |
  | --- | --- |
  |  |  |
  | --- | --- |
  | HierarchyBuilder.Method | The different types of distributing labels to children nodes |

- Overview
- Package
- Class
- Use
- Tree
- Deprecated
- Index
- Help

- Prev Package
- Next Package

- Frames
- No Frames

- All Classes
